# Trigger pSA predicting recurrence from positive choline PET/CT with prostate cancer after initial treatment

**DOI:** 10.18632/oncotarget.24318

**Published:** 2018-01-24

**Authors:** Junbao Wei, Hengzong Zhu, Xiaoli Liao

**Affiliations:** ^1^ Department of Therapeutic Radiology, Guangxi Autonomous Regional Cancer Hospital & Cancer Hospital of Guangxi Medical University, Nanning, 530021, China; ^2^ Department of General Medicine, Longan Hospital of Traditional Chinese Medicine, Nanning, 532700, China; ^3^ The Oncology Department, Guangxi Autonomous Regional Cancer Hospital & Cancer Hospital of Guangxi Medical University, Nanning, 530021, China

**Keywords:** prostate cancer, recurrence, choline PET/CT, PSA, meta-analysis

## Abstract

**Purpose:**

To assess the relationship between the diagnostic accuracy of Choline positron emission tomography/computed tomography (PET/CT) and the trigger prostate-specific antigen (PSA) level in patients with a biochemical recurrence of prostate cancer.

**Materials and Methods:**

A meta-analysis was conducted to synthesize data across multiple studies.

**Results:**

The pooled sensitivity and specificity of choline PET/CT were 82% (95% Confidence Interval (CI):80–84%) and 92% (95%CI: 90–93%), respectively. The pooled sensitivity and specificity of ^18^F-choline PET/CT were 81% (95%CI: 78–84%) and 90% (95%CI: 85–93%), respectively. The pooled sensitivity and specificity of ^11^C-choline PET/CT were 83% (95% CI: 80–86%) and 92% (95% CI: 90–94%), respectively. The pooled detection rate of ^18^F-choline PET/CT and ^11^C-choline PET/CT were 58% (95% CI: 48–68%) and 58% (95%CI: 49–68%), respectively.

**Conclusions:**

Trigger PSA is an important risk factor for positive findings of Choline PET/CT and the detection rate of Choline PET/CT for recurrent prostate cancer increased in parallel with raises in PSA-values. Choline PET/CT got higher detection rate while the trigger PSA > 2ng/ml.

## INTRODUCTION

Prostate cancer (PC) ranks second malignant tumor in male in developed world [1]. Radical prostatectomy or radiotherapy has succeeded in treating patients who suffered from localized PC. About 15% to 77% of patients suffered PSA relapse within 5 years after initial treatment [2–4]. High-intensity focused ultrasound ablation, brachytherapy, cryotherapy or radical prostatectomy (RP) have been successfully used to treat recurrent tumor, metastasis will occur in 3 years if these treatments are not performed in time [4, 5]. The most important issue before initial treatment is to identify if the illness is localized or metastasis since PSA relapse is still a clinical dilemma.

However, it is difficult to determine the presence of recurrence, since the specificity and sensitivity for recurrent prostate are poor of traditional imaging approaches such as CT or TRUS [6, 7]. Recently, 18F-choline PET/CT and ^11^C-choline PET/CT have been proved effective for detecting recurrent PC with PSA relapse [4], but the most accurate imaging tracer of choline PET/CT and the relation among diagnostic accuracy of choline PET/CT and trigger PSA are still controversial. The purpose of our study is to evaluate the diagnostic efficiency of choline PET/CT in identifying recurrent PC and estimate the connection between its diagnostic efficiency and the trigger PSA level in PSA relapse patients.

## RESULTS

### Search results and study selection

**Figure 1:**
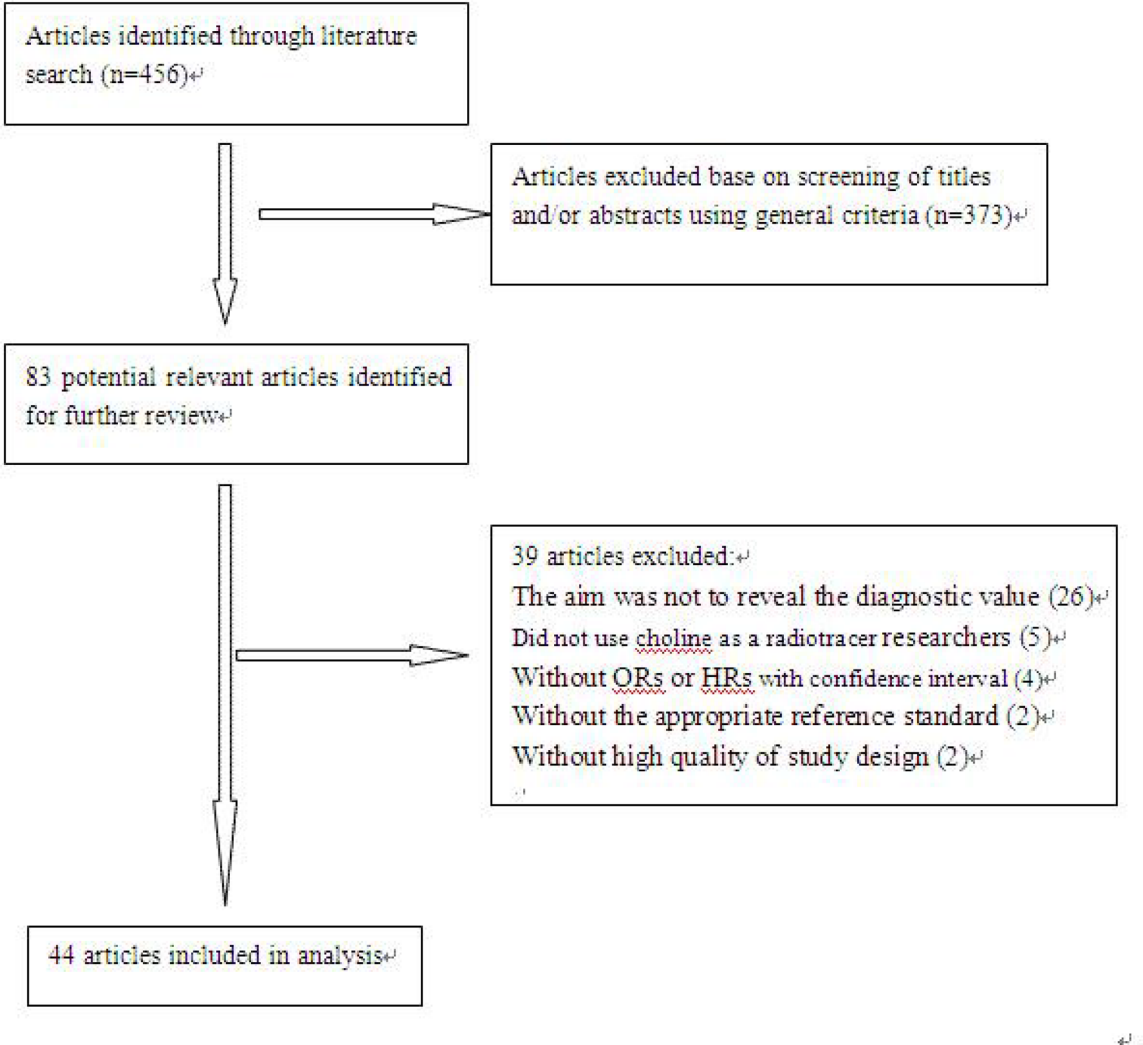
Flow chart for selection of eligible studies

The steps of the literature search and article screening were shown in Figure 1. 456 articles were found after preliminary online searches, 83 articles among them potentially met the inclusive standards after screening abstracts and titles, 39 of them were excluded after we study every full text carefully, the reasons are as follows: ①The articles was not to demonstrate the diagnostic value of choline PET/CT (*n* = 26); ② choline was not used as a imaging tracer (*n* = 5); ③ articles didn't provide enough data to identify or calculate TP, FP, TN, FN and/or detecting rate (*n* = 4); ④ histopathology and/or clinical and imagine follow up were not used as the reference standard (*n* = 2); ⑤ without high quality of study design (*n* = 2). After screening, 44 articles [15–21, 23–41, 43, 44, 46–61] were included in our meta-analysis.

### Study characteristics

Individual study characteristics are presented in Supporting Supplementary Table 1. As some lesion were deeply located and was technically and/or morally difficult to carry out histopathological analysis for all the pathological changes, it is unavoidable to apply histopathology and/or clinical and imaging follow up as reference standard [1].

### Data analysis

**Figure 2:**
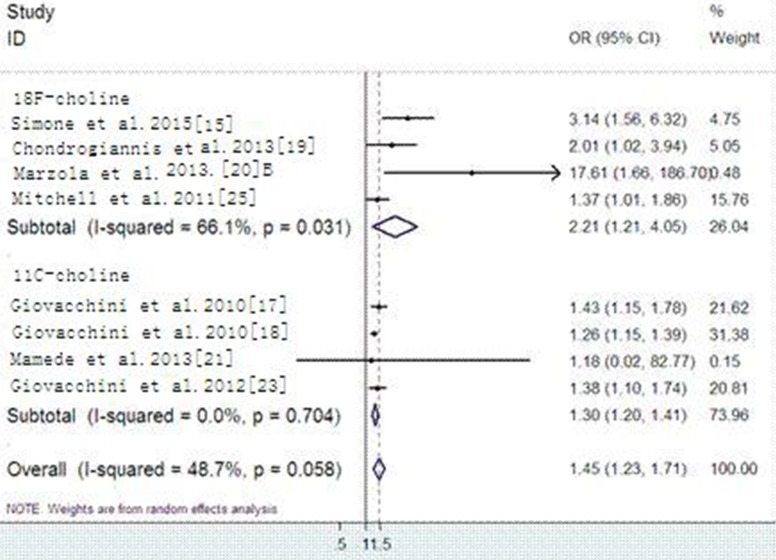
Forest plots of trigger PSA influencing 18F-choline PET/CT and C11-choline PET/CT detection rate (with subgroup analysis)

Nine sets of data along with eight studies reported the trigger PSA influencing Choline PET/CT detecting rate with PSA relapse PC. Among the nine sets of data, only one articles [21] showed that trigger PSA was not significantly (*P* = 0.938) related with positive choline PET/CT while the others showed significantly (*p* < 0.05 = influence. Heterogeneity of selected studies was examined according to the I^2^ statistic and *p*-value and obvious heterogeneity was found in the nine sets of data (*I^2^* = 58.8%, *p* = 0.013) with a combined OR of 1.25 (95%CI, 1.18–1.34) as calculated by the random-effects model. Sensitivity analysis was conducted to explore heterogeneity (Supplementary Figure 1). The major source of heterogeneity was from the data which was based on Mazola's study [20A] including 133 patients. No heterogeneity was found among the other studies when that set of data was excluded (*I^2^* = 48.7%, *p* = 0.058). The fixed-effects model was applied to calculate the OR of the remaining seven sets of data and the combined OR was 1.45 (95% CI, 1.23 = 1.71, Figure 2). After a subgroup analysis conducted by tracer, we found a combined OR of 2.21 (95%CI, 1.21–4.05) with obvious heterogeneity (I^2^ = 58.8%, *p* = 0.013) in ^18^F-choline PET/CT, while the combined OR is 1.30 (95%CI, 1.20–1.41) without heterogeneity (I^2^ = 0%, *p* = 0.704) in C11-choline PET/CT, both were calculated by the random-effects model (Figure 2).

**Figure 3:**
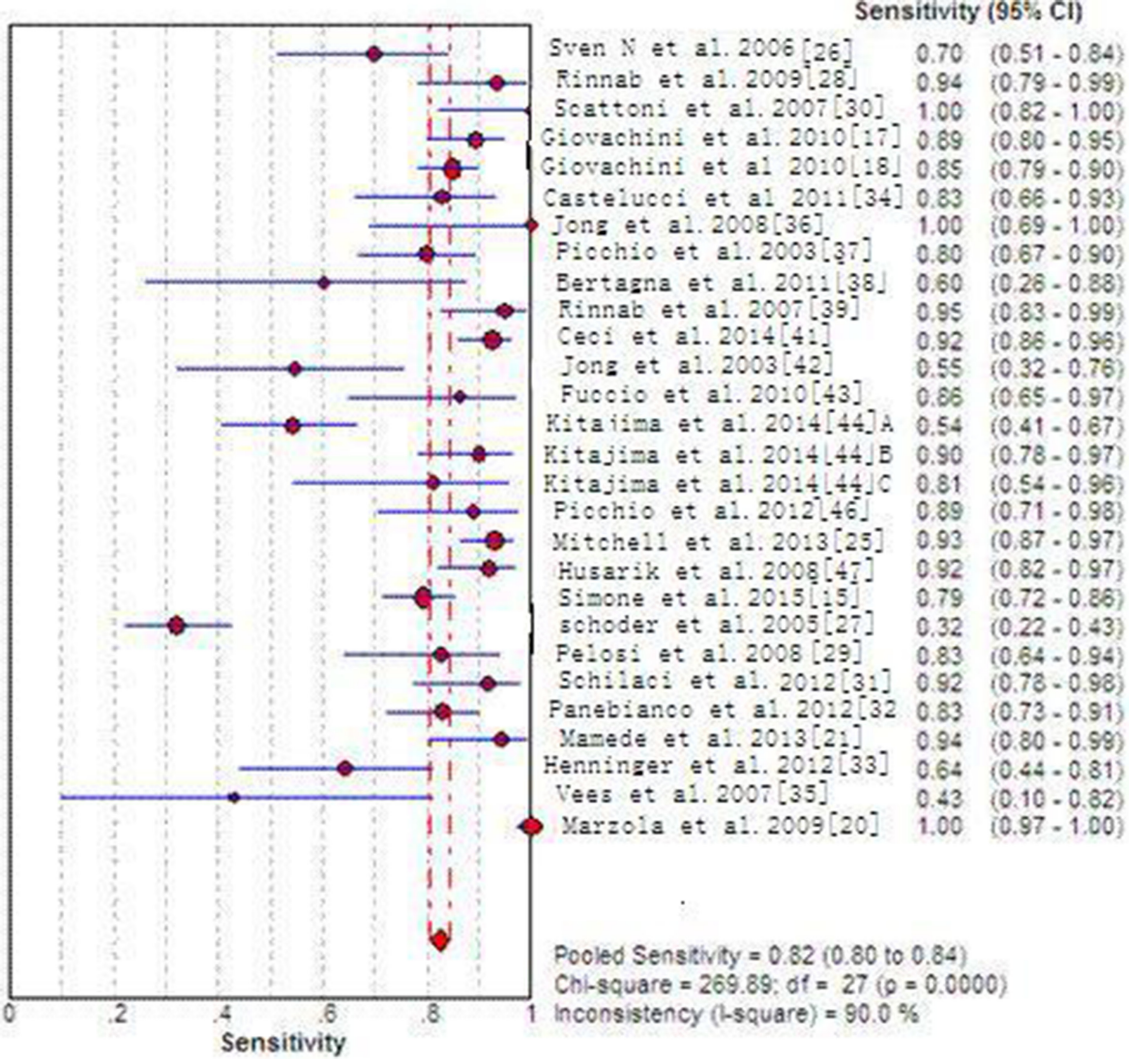
Forest plot of pooled sensitivity of choline PET/CT

**Figure 4:**
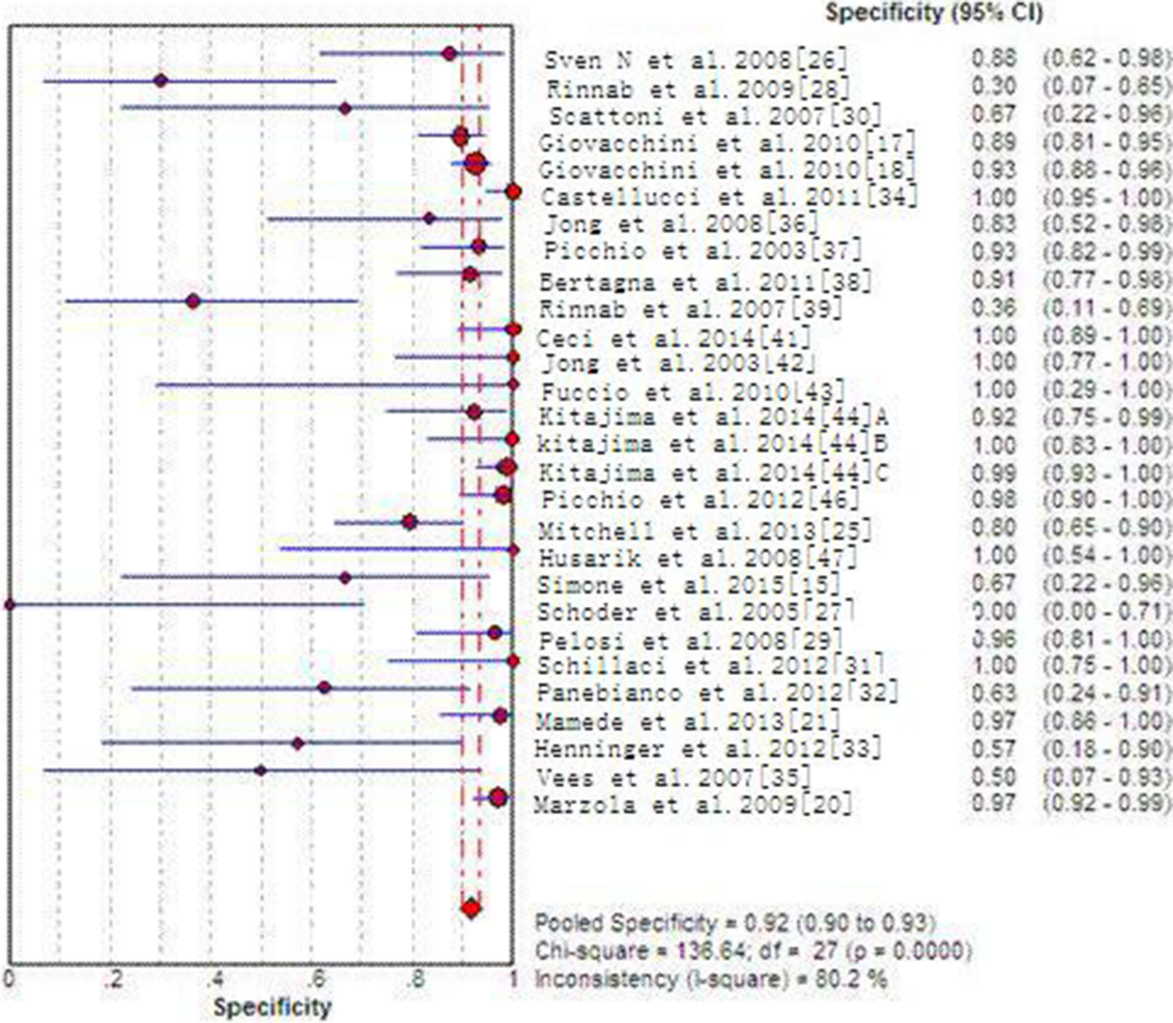
Forest plot of pooled specificity of choline PET/CT

Across all the 27 included articles with 29 sets of data which provide enough data to identify or calculate TP, FP, TN and FN, specificity, sensitivity, positive/negative predictive value and DOR for each individual study are listed in Supporting Supplementary Table 2. 29 sets of data indicate pool sensitivity and specificity of 82% (95%CI: 80–84%) and 92% (95% CI 90–93%), respectively. The forest plots are shown in Figures 3–4. The AUC of choline PET/CT is 0.9264, the Q^*^ index estimate of is 0.8609 (Supporting Supplementary Figure 2). In a subgroup analysis (Table 1), tracer type (^18^F-choline VS. ^11^C-choline) was compared with specificity, sensitivity, PLR, NLR and DOR, there were no significant differences between the two type of choline PET/CT (*P* > 0.05).

**Table 1:** Sensitivity, specificity, positive and negative predictive value and DOR of 11C-choline PET/CT and ^18^F-Choline PET/CT

Subgroup	Summary sensitivity (95% CI)	Summary specificity (95% CI)	Summary LR+ (95% CI)	Summary LR– (95% CI)	DOR (95% CI)
Overall	0.82 (0.80–0.84)	0.92 (0.90–0.93)	6.61 (3.46–12.61)	0.20 (0.14–0.28)	38.55 (19.83–74.94)
^11^C–choline	0.83 (0.80–0.86)	0.92 (0.90–0.94)	8.13 (3.95–16.71)	0.21 (0.14–0.30)	47.79 (25.93–88.10)
^18^F–choline	0.81 (0.78–0.84)	0.90 (0.85–0.93)	4.71 (1.25–17.82)	0.20 (0.10–0.42)	25.78 (5.59–118.93)
*P*	0.532	0.220	0.349	0.305	0.370

36 articles reported the detecting rate result and the pool detecting rate was 59% (95% CI 51–66%) with high heterogeneity (*p* < 0.000, *I^2^* = 96.1%). In a subgroup analysis, the overall detecting rate of 11C-choline PET/CT and 18F-Choline PET/CT are 59% and 58%, respectively (Figures 5–6). Summary detecting rates of two type of Choline PET/CT with different trigger PSA value were showed in Table 2 and Supporting Supplementary Figures 3–18. After corresponding comparing the two type of Choline PET/CT with different PSA thresholds, there were no significant differences between the two type of choline PET/CT (*P* > 0.05).

**Figure 5:**
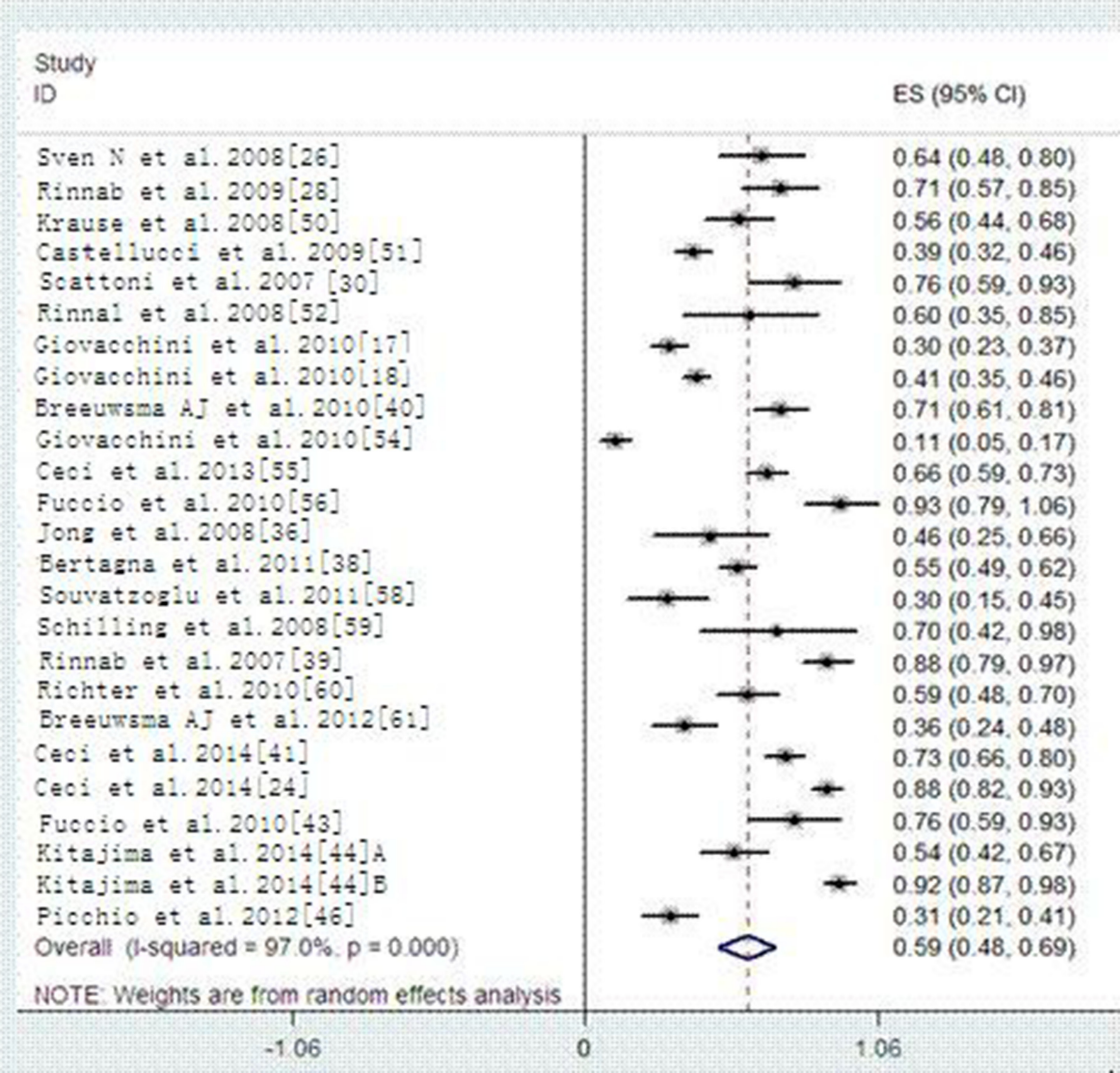
Detected rate of 11C-Choline PET/CT

**Figure 6:**
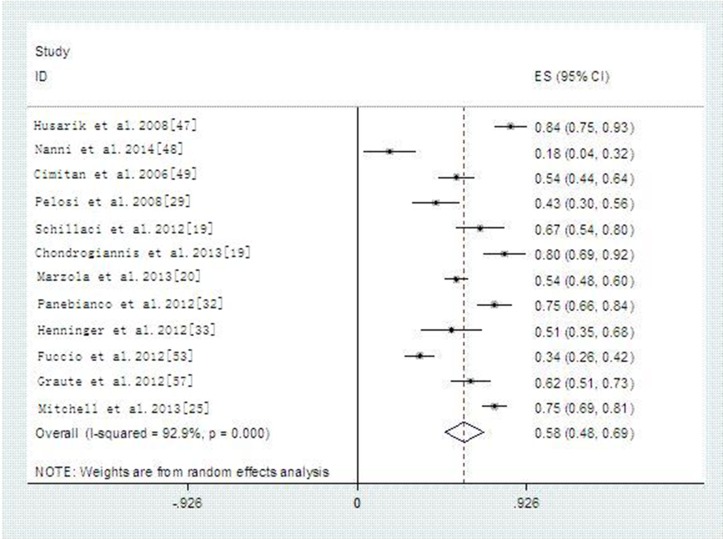
Detected rate of 18F-Choline-PET/CT

**Table 2:** Detected rate of ^11^C-choline PET/CT and ^18^F-Choline PET/CT with different trigger PSA

Subgroup	< 0.5ng/ml	< 1 ng/ml	< 1.5 ng/ml	> 2ng/ml	> 3ng/ml	> 4ng/ml	> 5 ng/ml	> 10 ng/ml	Total
	95%CI	95%CI	95%CI	95%CI	95%CI	95%CI	95%CI	95%CI	95%CI
^11^C-choline	8%	18%	26%	73%	80%	85%	69%	86%	59%
	1%-15%	9%-28%	20%-33%	63%-83%	73%-88%	73%-97%	54%-84%	79%-94%	49% – 69%
^18^F-Choline	25%	39%	33%	83%	80%	93%	82%	84%	58%
	18%-32%	12%-65%	25%-42%	76%-90%	64%-96%	84%-101%	71%-93%	74%-95%	48% – 69%
*P*	0.090	0.183	0.946	0.160	0.858	0.449	0.539	0.283	0.680

### Quality evaluation

Each study included in our meta-analysis fulfilled over 8 of the 14 QUADAS criteria for quality evaluation, the quality of the included studies was acceptable (Supporting Supplementary Table 3).

## DISCUSSION

It is critical to identify local or systemic tumor for the treatment of PC patients [29]. PET/CT imaging has become a popular topic recently, but 18FDG has been proved to be limited value with poor sensitivity [8, 22, 42, 45]. Recently, some centers also carry out PET/ CT with ^18^F-choline or ^C^11-choline radiotracer, the results are still contentious, even there have been several researches reported ^11^C-choline and ^18^F-choline were promising alternative radiotracers which can provides valuable information not only on local regional but also distant sites of recurrence. These researches also imply a connection between trigger PSA values and imaging sensitivity [2].

Some different studies reported the diagnostic efficiency are still controversial of choline PET/CT detecting recurrence PC. Most of the studies indicates that choline PET/CT is accurate in diagnosis recurrence PC [18, 36, 20, 21]. However, Vees [37] stated the specificity and sensitivity of ^18^F-choline PET/CT are both poor (50% and 43%, respectively). Henninger [34] also got a lower specificity and sensitivity of 57.1% and 64.3%. For another imaging tracer 11C, Jong [47] and Bertagna [48] informed the sensitivity of 55% and 60%, respectively; Rinnab [28] and Reske [50] informed the specificity of 36% and 66.6%, respectively. Across the 28 included articles which can calculate the specificity and sensitivity, a pooled specificity and sensitivity of 92% (95% CI 90–93%) and 82% (95% CI 80–84%) were found in our Meta-analysis. Our subgroup analysis informed that the pooled sensitivity of ^18^F-choline PET/CT and C11-choline PET/CT are 81% and 83%, respectively; the pooled specificity of ^18^F-choline PET/CT and C11-choline PET/CT are 90% and 92%, respectively. Because no significant differences were found in specificity, sensitivity, NLR, PLR and DOR between them, they have no obvious difference in detecting recurrent PC.

In our study, Choline PET/CT has good diagnostic accuracy on detecting recurrent PC across the 28 included articles which can calculate the sensitivity and specificity, on the contrary, the overall detecting rate was only 59% in 37 included studies which can calculate the detection rate, in the subgroup analysis, the overall detecting rate of ^18^F-choline PET/CT and C11-choline PET/CT are 58% and 59%, respectively. Eight studies along with nine sets of data reported the trigger PSA influencing Choline PET/CT detecting rate with PSA relapse PC. Only one article [21] showed that trigger PSA was not an important influence factor for positive findings of Choline PET/CT (OR = 1.182, 95%CI:0.017–82.774, *P* = 0.938) while the others got the contrary result. A combined OR of 1.25 for trigger PSA predicting recurrence from choline PET/CT positive findings with biochemical failure prostate cancer after initial treatment was found in our meta-analysis, after a subgroup analysis, the combined OR were 2.21 and 1.45 in ^18^F-choline and ^C^11-choline PET/CT, respectively. This overall low detection rate is because of a correlation between trigger PSA and the Choline PET/CT detecting rate, a higher trigger PSA value means a higher positive rate of choline PET/CT.

However, not so much data is available on Choline PET/CT detecting rate for the patients who suffered PSA relapse with lower PSA values [3]. Krause's result [2, 50] showed a detecting rate of recurrence of 56% of ^11^C-choline PET/CT while the mean PSA is 5.9 ng/ml. They also reported a linear relationship between detecting rate and trigger PSA: the detecting rate are 36%, 43%, 62% and 73% while the PSA value are < 1 ng/ml, 1 to 2 ng/ml, 2 to 3 ng/ml and > 3 ng/ml, respectively. With the same imaging tracer of 11C when the trigger PSA < 1.5 ng/ml, Castellucci and Rannab reported the large difference detecting rates of 21% and 53.8%, respectively [5]. With the other imaging tracer of ^18^F when the trigger PSA < 1 ng/ml, Simone and Schillaci reported the large difference detecting rates of 76% and 20%, respectively [15, 31].

In our meta-analysis, the pooled detecting rate of 11C-choline PET/CT are 8%, 18% and 26% while the trigger PSA are < 0.5ng/ml, < 1 ng/ml and < 1.5 ng/ml, respectively. The pooled detecting rate of 18F-choline PET/CT are 25%, 39% and 33% while the trigger PSA are < 0.5ng/ml, < 1 ng/ml and < 1.5 ng/ml, respectively. For all lower PSA values, the detecting efficiency of the two type of C-choline PET/CT are both lower. While the trigger PSA improve to > 2 ng/ml, > 3 ng/ml, > 4 ng/ml, > 5 ng/ml and > 10 ng/ml, the pooled detecting rate of ^C^11-choline PET/CT are 73%, 80%, 85%, 69% and 86%, respectively, the pooled detecting rate of ^18^F-choline PET/CT are 83%, 80%, 93%, 82% and 84%, respectively. For all higher PSA values, the detecting efficiency of the two type of C-choline PET/CT are both higher. In that case, the linear relationship doesn't exist between trigger PSA and detecting rate when the trigger level > 2ng/ml.

Our study still has some limitations. Just like all the meta-analysis of diagnostic accuracy, our study is also restricted by the degree of heterogeneity which consists of methodological quality, radiologist experience and approach to image interpretation. We deal with the heterogeneity issue in 3 ways: (1) Strict inclusive criteria were used to minimize diversity while selecting studies, (2) Provided objective and rigorous evaluating of quality of included articles by a validated tool (QUADAS), (3) Performed stratified analysis base on the factors that probably lead to heterogeneity. Another limitation is that there is no acceptable gold standard, which is general weakness of most studies researching different tumors and various imaging modality for diagnostic efficiency in the diagnosing recurrent lesions. As the lesion were deeply located and was technically and morally difficult to carry out histopathological analysis for all the pathological changes, it is unavoidable to apply histopathology and/or clinical and imaging follow up as reference standard, therefore there is a chance for verification bias [1]. At last, heterogeneity also can due to some unreported or unmeasured study features, which is inherent to a meta-analysis based on published data.

## MATERIALS AND METHODS

### Search strategy and definitions

### Data extraction and quality assessment

The following data were collected from each publication: the name of the first author, year of publication, study design, No. of patients, PSA level, initial treatment type, reference standard used, criteria used to define the cut-off between positive and negative on choline PET/CT, sensitivity, specificity, PPV, NPV, overall accuracy, detection rate and ORs or HRs with confidence interval. Sensitivity and specificity were used when the standards of reference for discriminating true from false findings were pathological findings and/or a composite yet acceptable surrogate including other imaging methods (CT, MR, BS), clinical follow-up for at least 12 months, including repeated CT, MR, BS and choline PET/CT revealing the appearance of further metastatic lesions, or the disappearance of metastatic lesions associated with normalization of PSA values (< 0.2 ng/ml) following systemic therapy. Detection rate was defined as the number of subjects positive on choline PET/CT in relation to the overall number of subjects included in the study and Detection rate was used when the examinations used as the standard of reference including other imaging methods were carried out at the same time as the index examination. The OR was assumed to be the same as HR, and all results are reported as OR for simplicity [8]. Concerning to the quality of study design, study quality was assessed with the QUADAS checklist for studies of diagnostic accuracy included in systematic reviews [9], only the article in which the number of the answer ‘yes’ for the 14 questions in QUADAS quality assessment tool was larger than 9 was included.

### Statistical analysis

Statistical heterogeneity among studies was assessed using the chi-square test (results were defined as heterogeneous for a *P* value < 0.10), and the potential inconsistency was quantified through the I2 statistic, which describes the percentage of total variation across studies that is due to heterogeneity rather than chance [10]. P < 0.05 was considered as having apparent heterogeneity for chi-squared tests and a random effect model was used for the primary meta-analysis to obtain summary estimates with 95% confidence intervals if heterogeneity existed.

For the articles which provide data that could be used to construct or calculate true-positive, false-positive, true-negative, and/or false-negative results, we calculated pooled specificity and pooled sensitivity for each modality. A value of 0.5 was added to all cells of studies that contained a count of zero to avoid potential problems in odds calculations for studies with specificities or sensitivities of 100%. Likelihood ratios (LR) are metrics that combine both specificity and sensitivity in their calculation. LR- is defined as the ratio of (1- sensitivity) over specificity whereas LR+ is defined as the ratio of sensitivity over (1- specificity). Both LRs equal 1 when there is absolutely no discriminating ability for a diagnostic test. Although there is no absolute standard, a good diagnostic test may have LR+ greater than 5.0 and LR- less than 0.2 [11]. Since the area under the ROC curve (AUC) was used as an alternative global measure of test performance [12], we also used the derived estimates of sensitivity, specificity and respective variances to construct a summary receiver operating characteristic (ROC) curve and the Q^*^ index. Then we did Z test to find whether the sensitivity, specificity and DOR and of ach technique were significantly different from the others. If *P* < 0.05, the result was considered to be statistically significant.

For the articles which provide ORs or HRs with confidence interval, if the heterogeneity was acceptable (I^2^ < 50%), a fixed effects analysis was conducted to calculate the pooled OR. In addition, a random effects model was used. The causes of heterogeneity were investigated by subgroup analyses. The detection rates were pooled using the generic inverse variance approach in the random-effects model [13].

Publication bias was assessed by using a scatter plot of the inverse of the square root of the effective sample size (ESS1/2) versus the diagnostic log odds ratio visually, which would have a symmetric funnel shape when publication bias was absent. Formal testing for publication bias was conducted by using a regression of the diagnostic log odds ratio against ESS1/2 and weighting according to the effective sample size, with *P* < 0.05 indicating significant asymmetry [14]. All statistical tests were 2-sided.

## CONCLUSIONS

18F and 11C choline PET/CT are accurate to diagnose recurrent lesion in PSA relapse patients for PC, their diagnostic efficiency was not significantly different. Trigger PSA is an important influence factor for positive findings of Choline PET/CT, the detecting rate getting higher in parallel with raises in PSA values. Choline PET/CT got higher detecting rate while the trigger PSA > 2 ng/ml.

### Ethical approval

This article does not contain any studies with human participants or animals performed by any of the authors. This is a diagnostic study.

### Disclosure

The scientific guarantor of this publication is Chunhua Wu. The authors of this manuscript declare no relationships with any companies, whose products or services may be related to the subject matter of the article. The authors state that this work has not received any funding. No complex statistical methods were necessary for this paper.

## SUPPLEMENTARY MATERIALS FIGURES AND TABLES




