# Supplementary material for: Trigger pSA predicting recurrence from positive choline PET/CT with prostate cancer after initial treatment
Source: Oncotarget. 2018 Jan 24;9(18):14630–41. doi: 10.18632/oncotarget.24318 (PMC5865695; doi:10.18632/oncotarget.24318)
Supplement: Supplementary file 1 [file oncotarget-09-14630-s001.pdf]

Trigger pSA predicting recurrence from positive choline PET/CT with prostate cancer after initial treatment

SUPPLEMENTARY MATERIALS

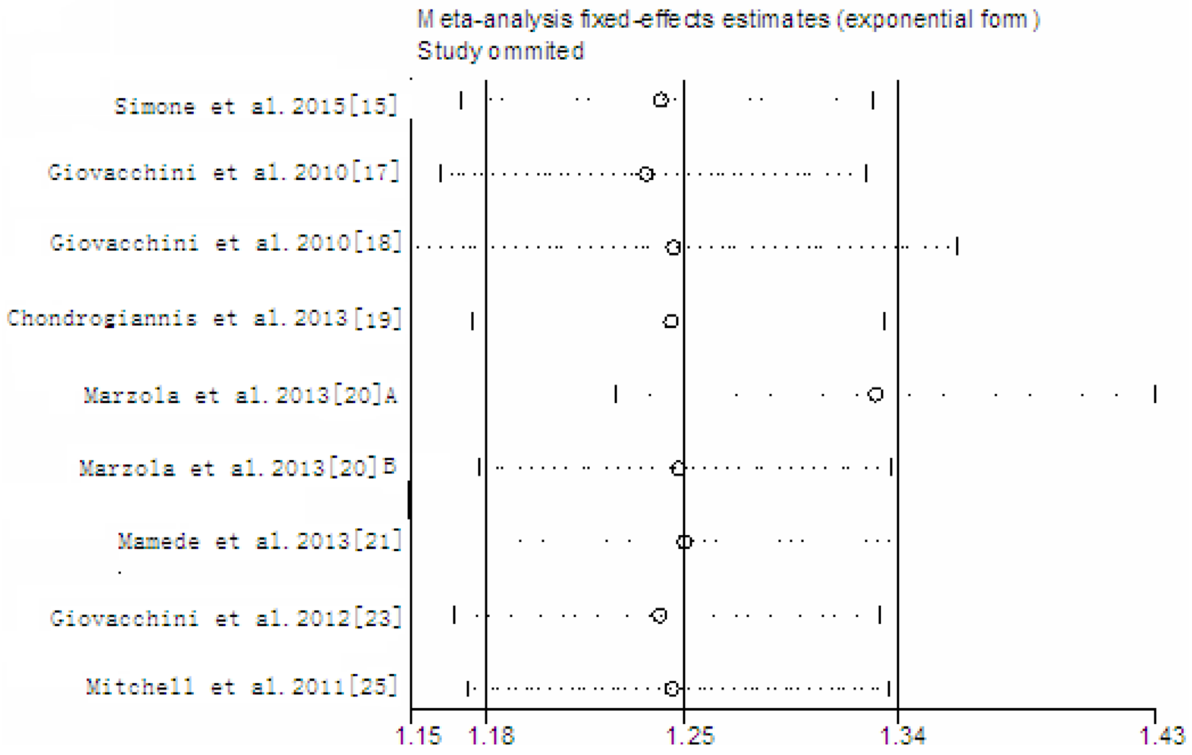

Supplementary Figure 1: Sensitivity analysis for combined ORs evaluating trigger PSA influencing Choline PET/CT detection rate.

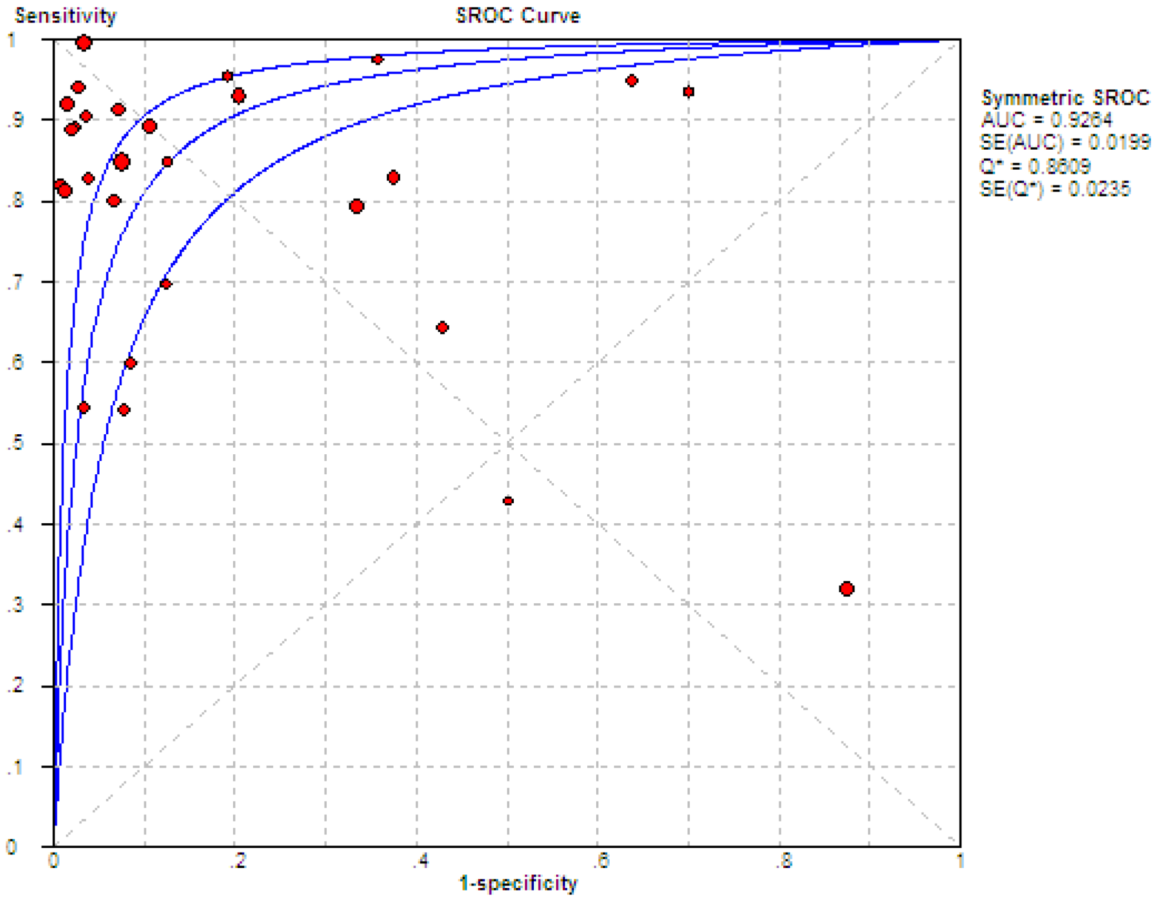

Supplementary Figure 2: Summary receiver operating characteristic (ROC) curves for choline PET/CT.

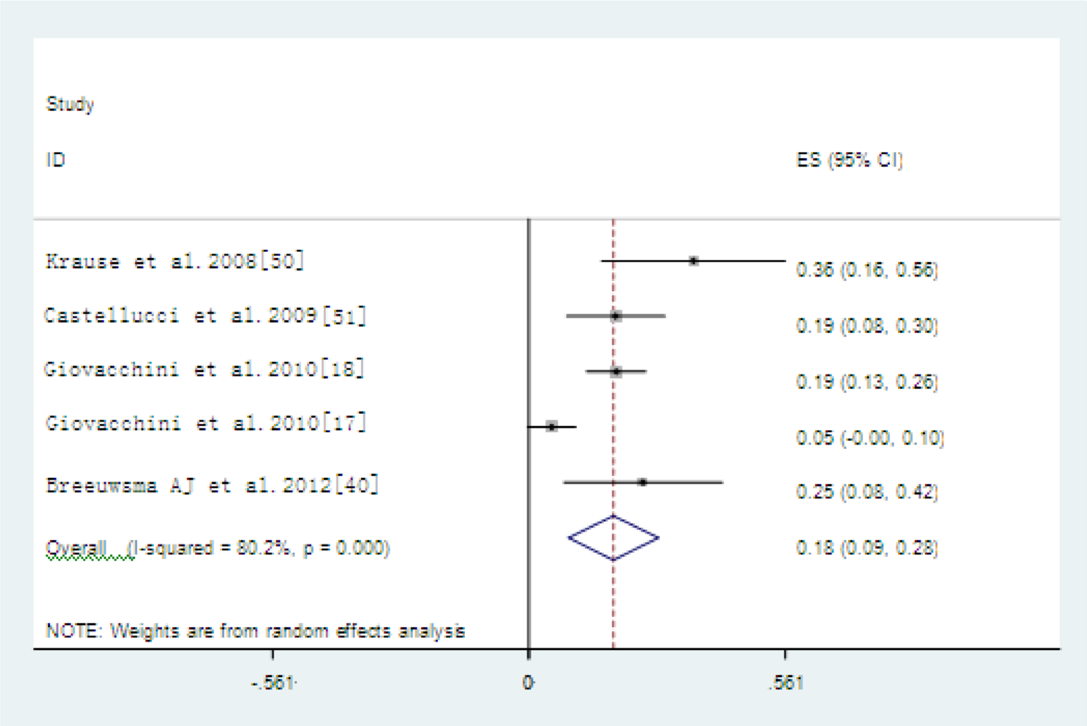

Supplementary Figure 3: Detected rate 11C-Choline PET/CT while PSA < 1 ng/ml.

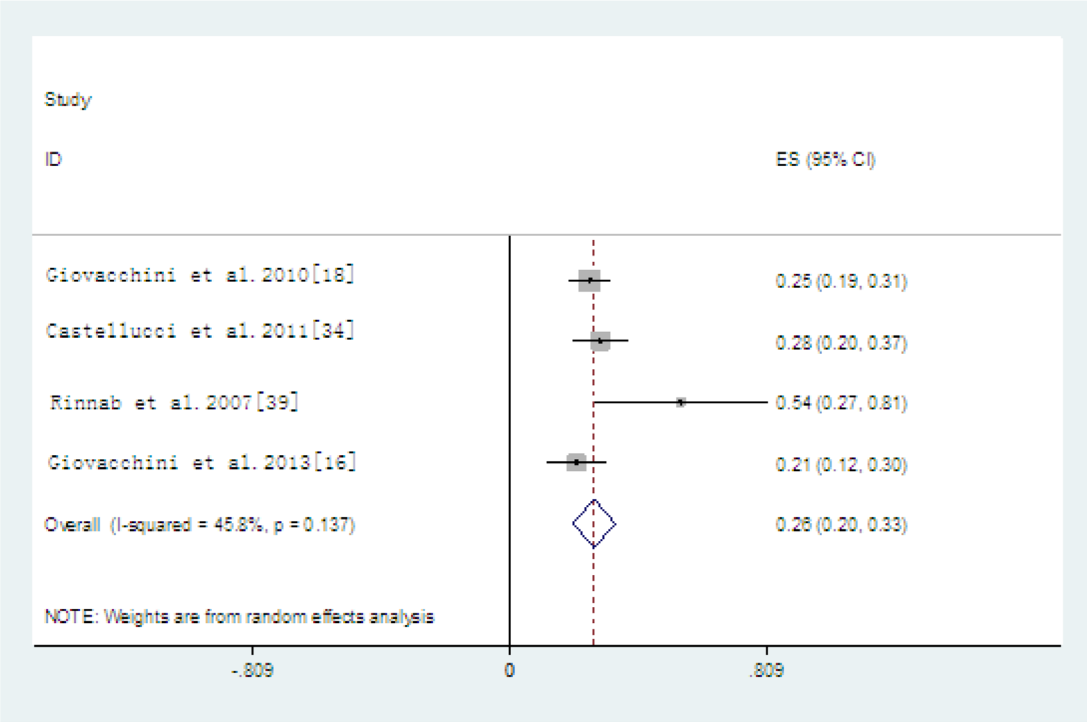

Supplementary Figure 4: Detected rate 11C-Choline PET/CT while PSA < 1.5 ng/ml.

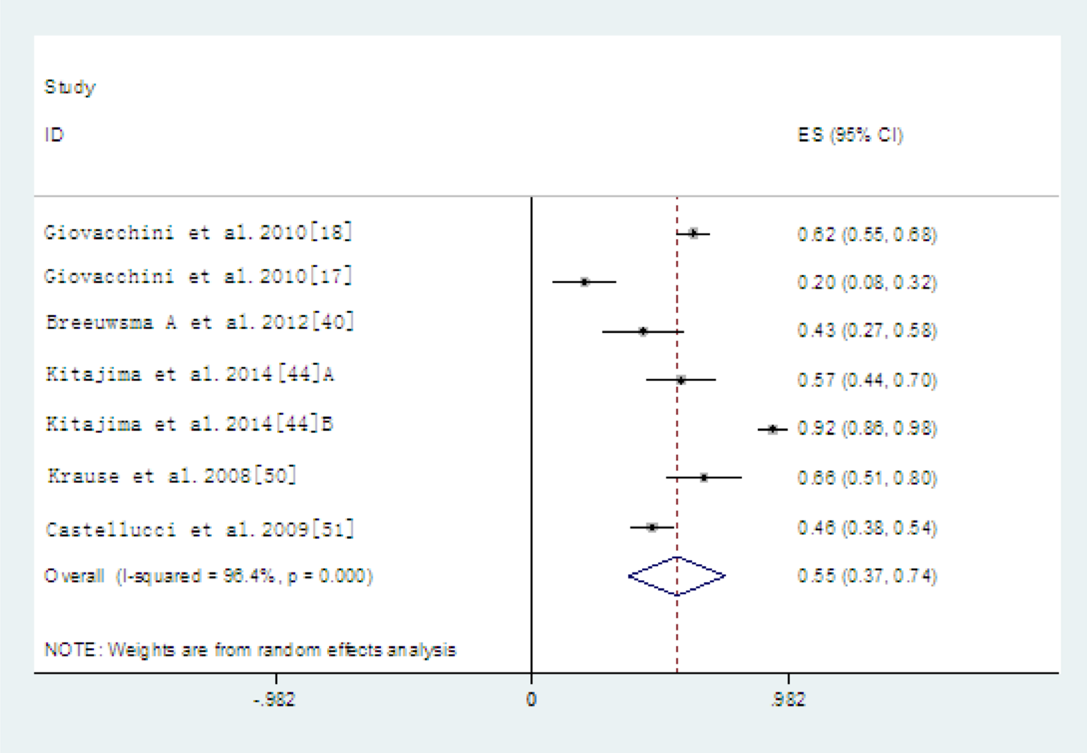

Supplementary Figure 5: Detected rate 11C-Choline PET/CT while PSA > 1 ng/ml.

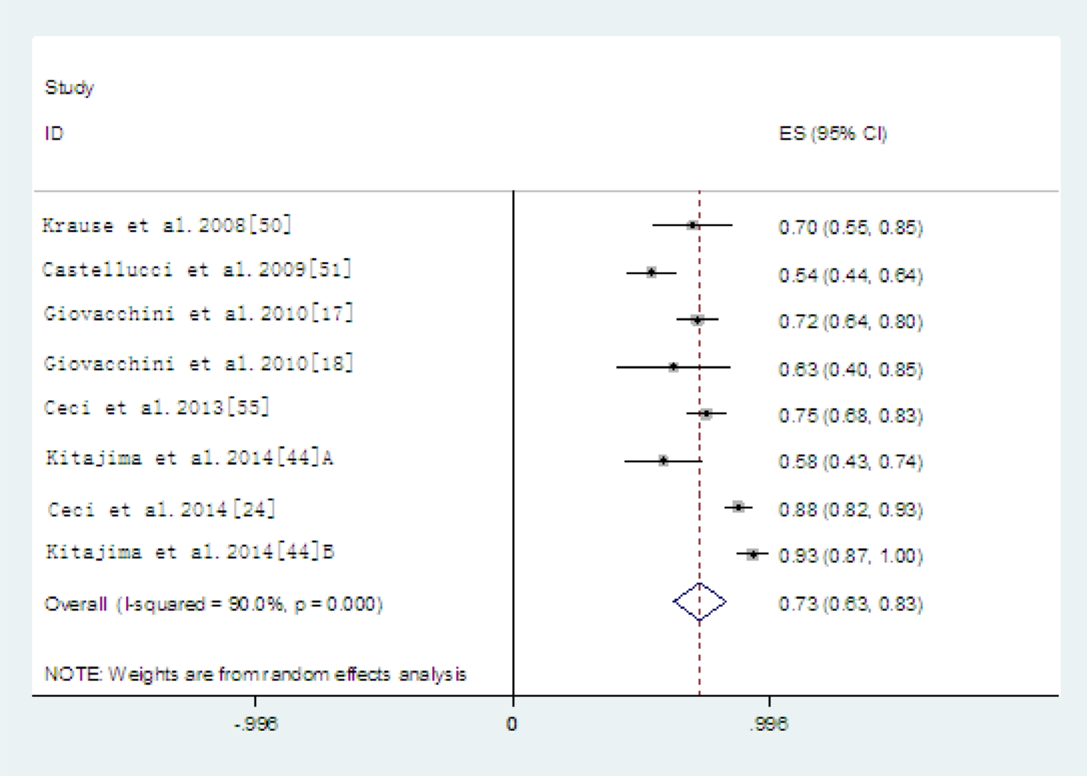

Supplementary Figure 6: Detected rate 11C-Choline PET/CT while PSA > 2 ng/ml.

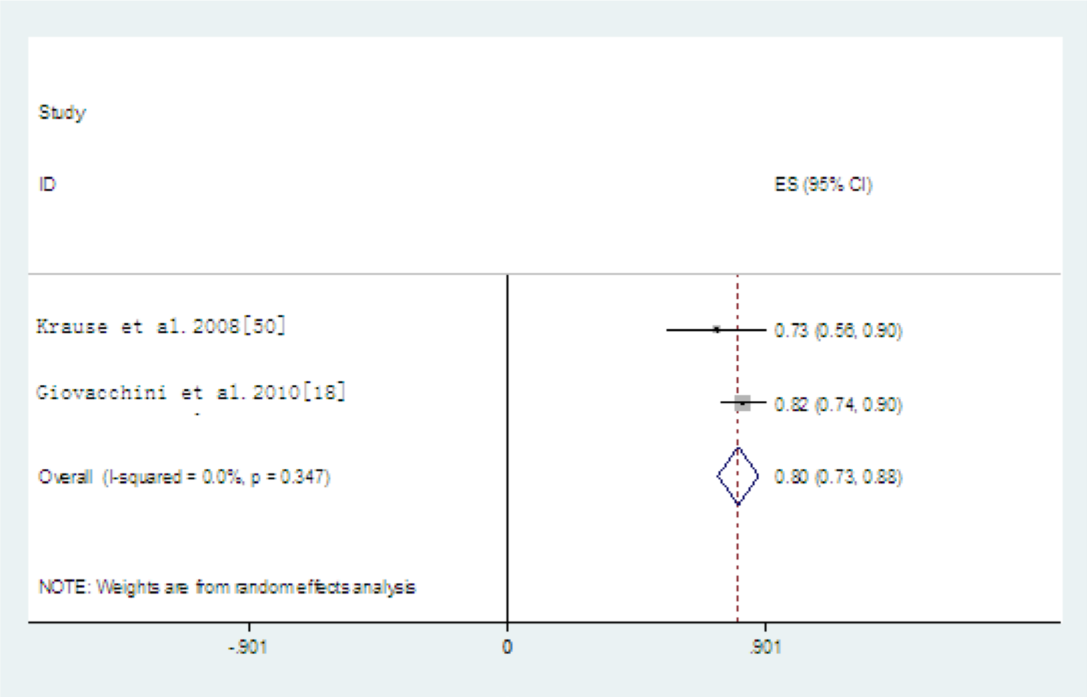

Supplementary Figure 7: Detected rate 11C-Choline PET/CT while PSA > 3 ng/ml.

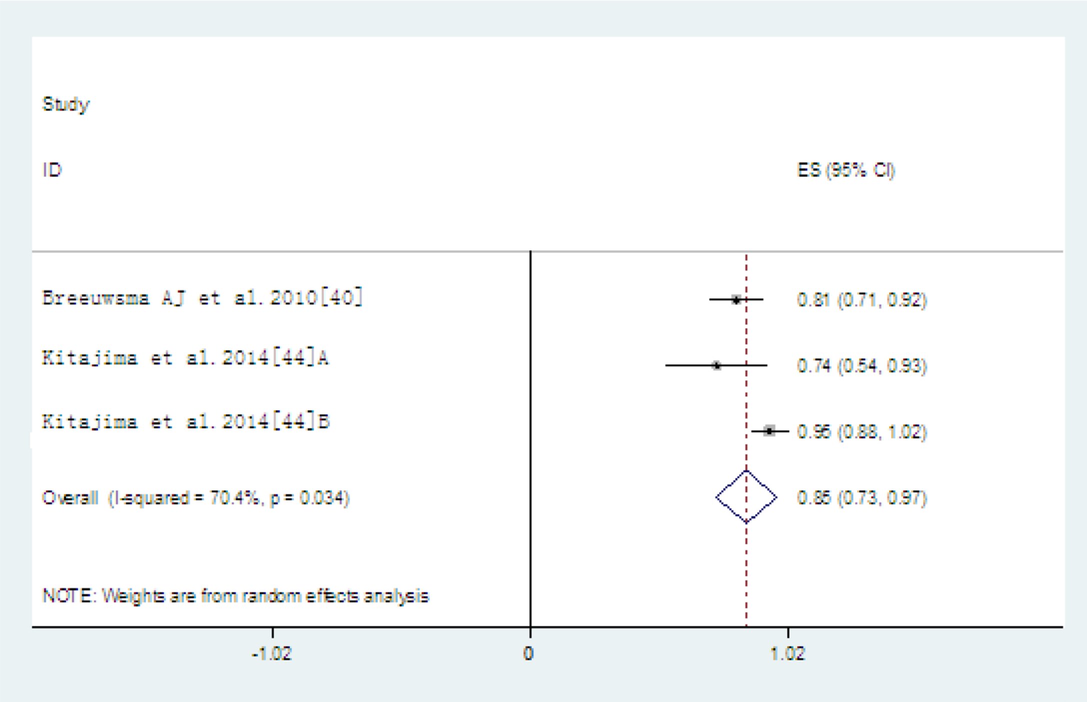

Supplementary Figure 8: Detected rate 11C-Choline PET/CT while PSA > 4 ng/ml.

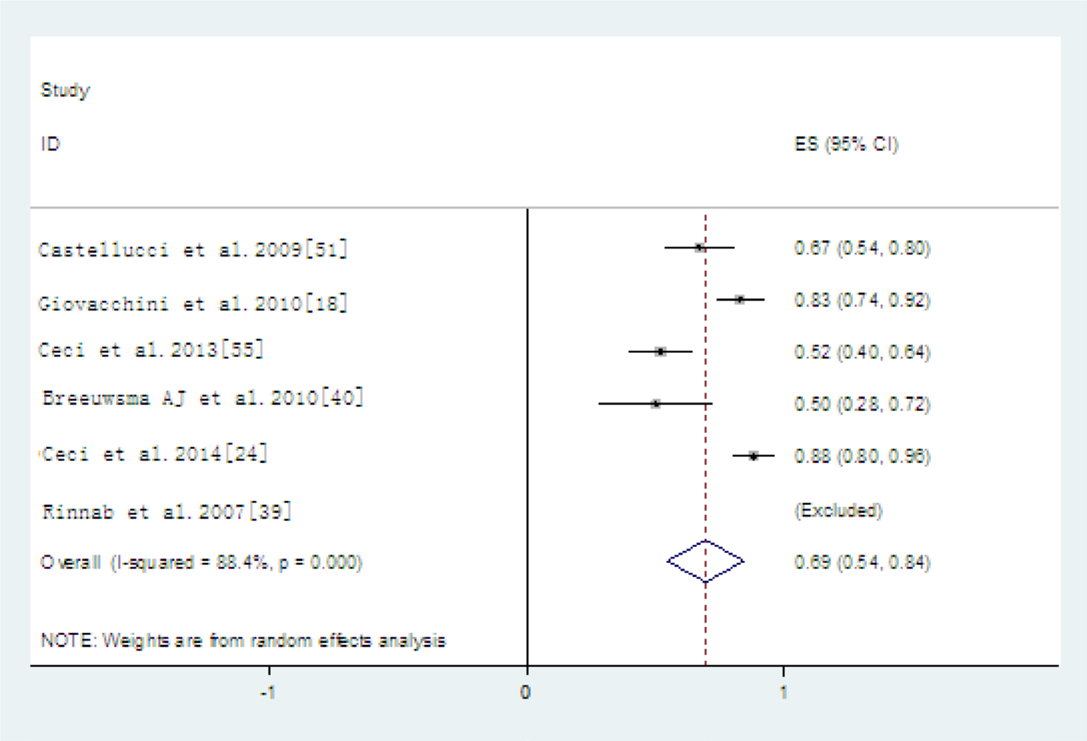

Supplementary Figure 9: Detected rate 11C-Choline PET/CT while PSA > 5ng/ml.

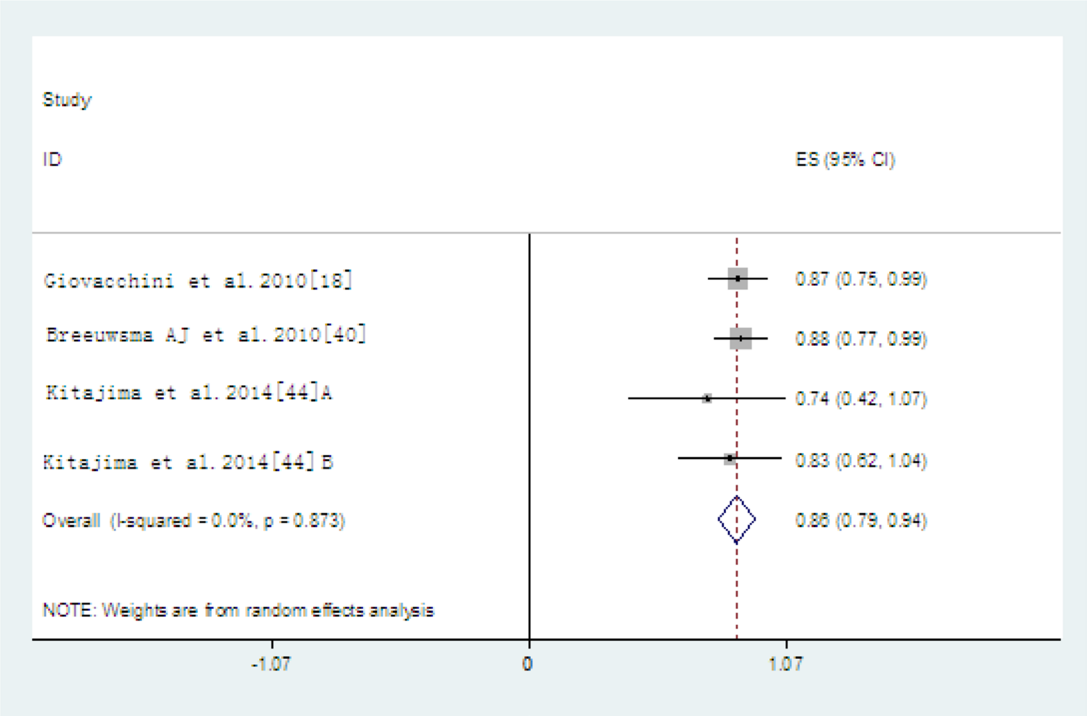

Supplementary Figure 10: Detected rate 11C-Choline PET/CT while PSA > 10ng/ml.

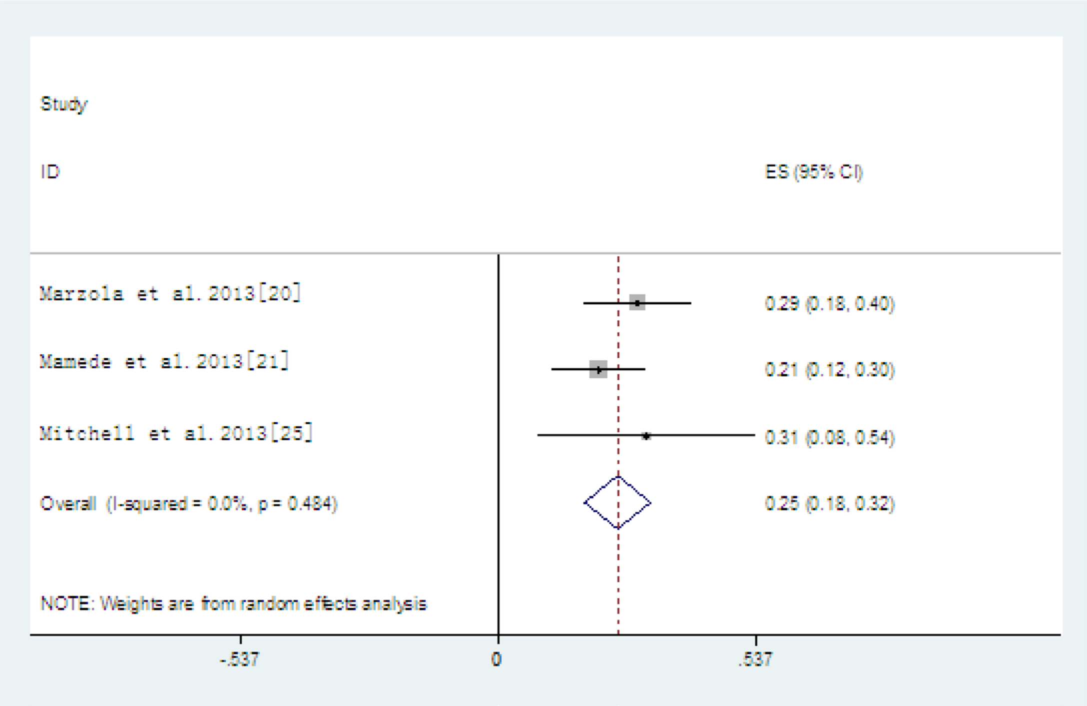

Supplementary Figure 11: Detected rate 18F-Choline PET/CT while PSA < 0.5 ng/ml.

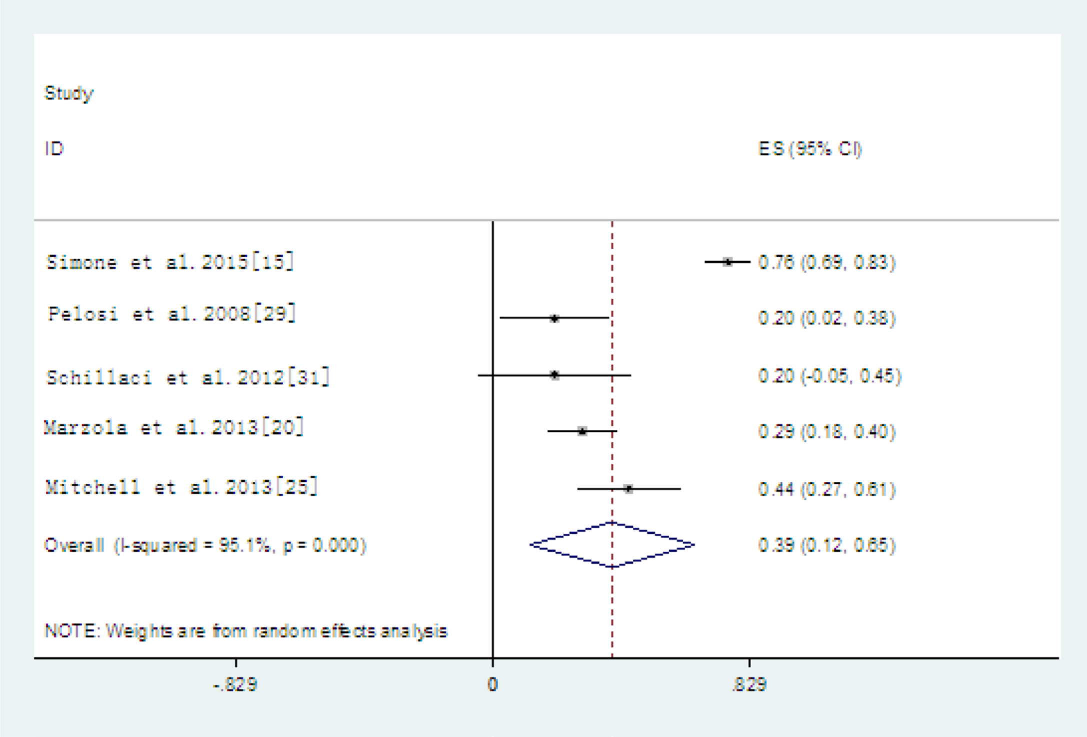

Supplementary Figure 12: Detected rate 18F-Choline PET/CT while PSA < 1 ng/ml.

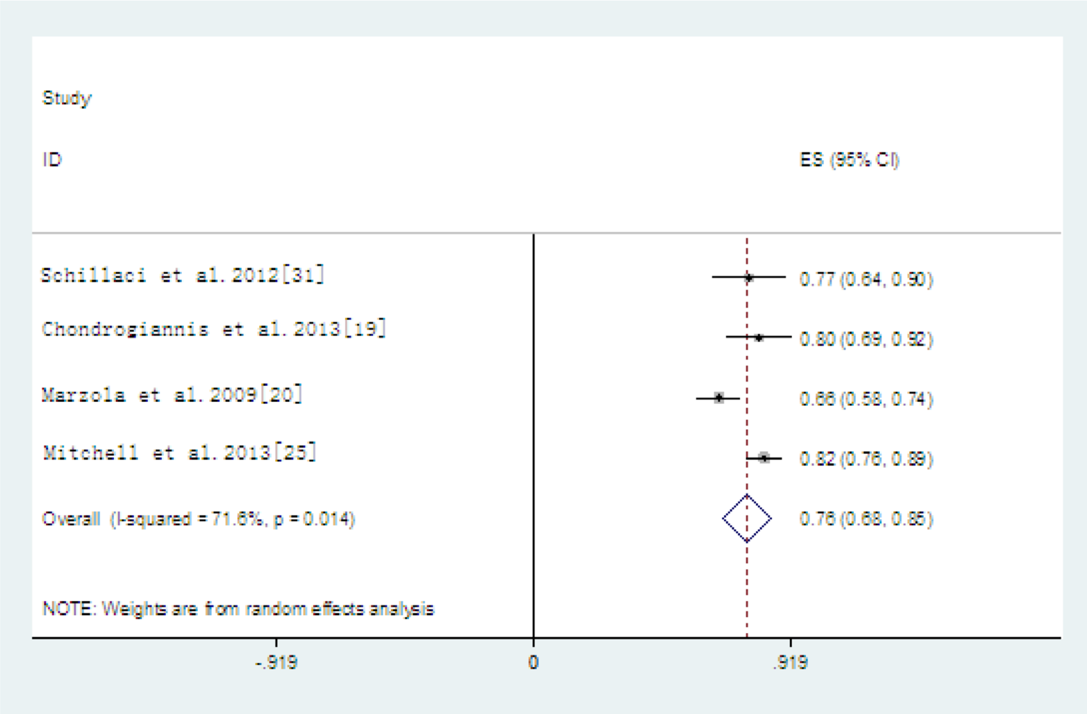

Supplementary Figure 13: Detected rate 18F-Choline PET/CT while PSA > 1 ng/ml.

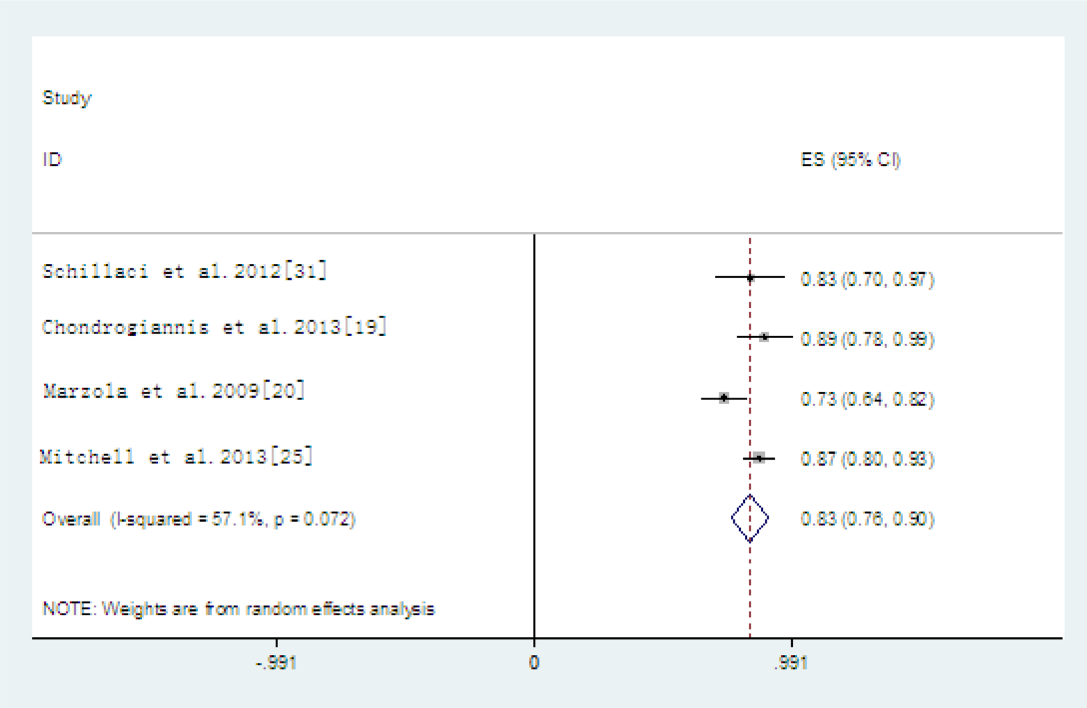

Supplementary Figure 14: Detected rate 18F-Choline PET/CT while PSA > 2 ng/ml.

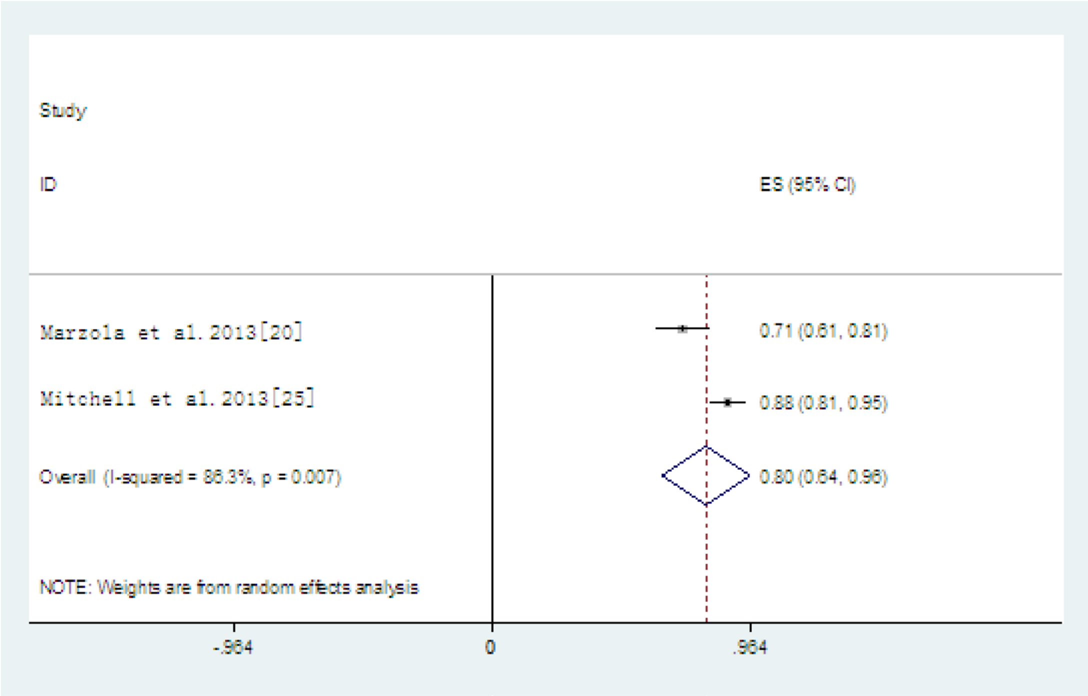

Supplementary Figure 15: Detected rate 18F-Choline PET/CT while PSA > 3 ng/ml.

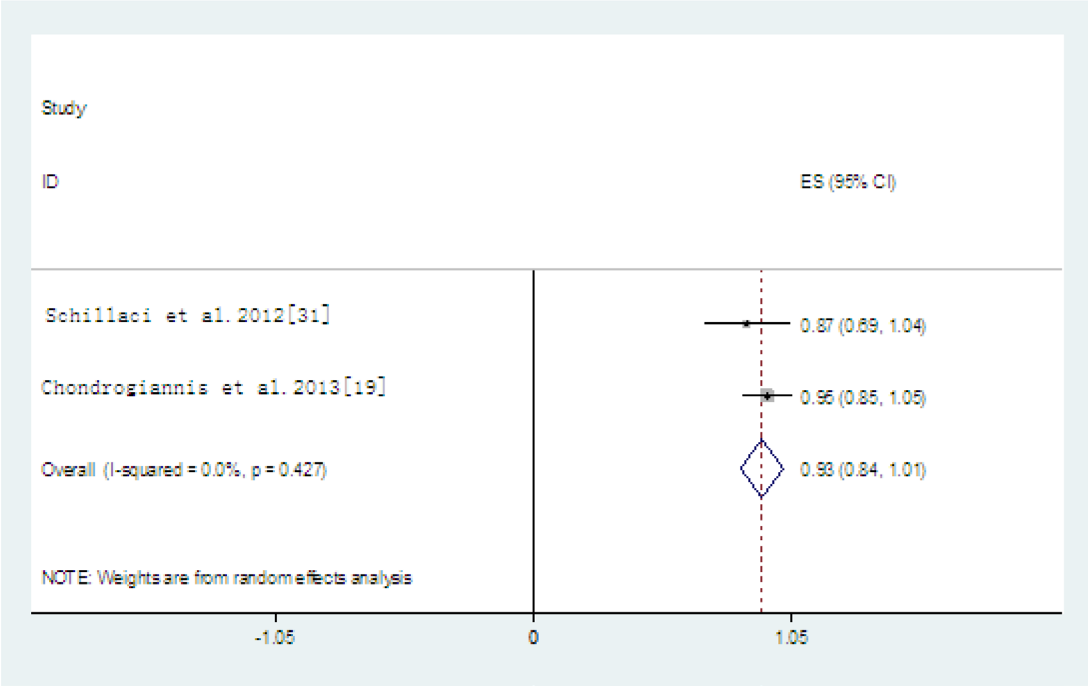

Supplementary Figure 16: Detected rate 18F-Choline PET/CT while PSA > 4 ng/ml.

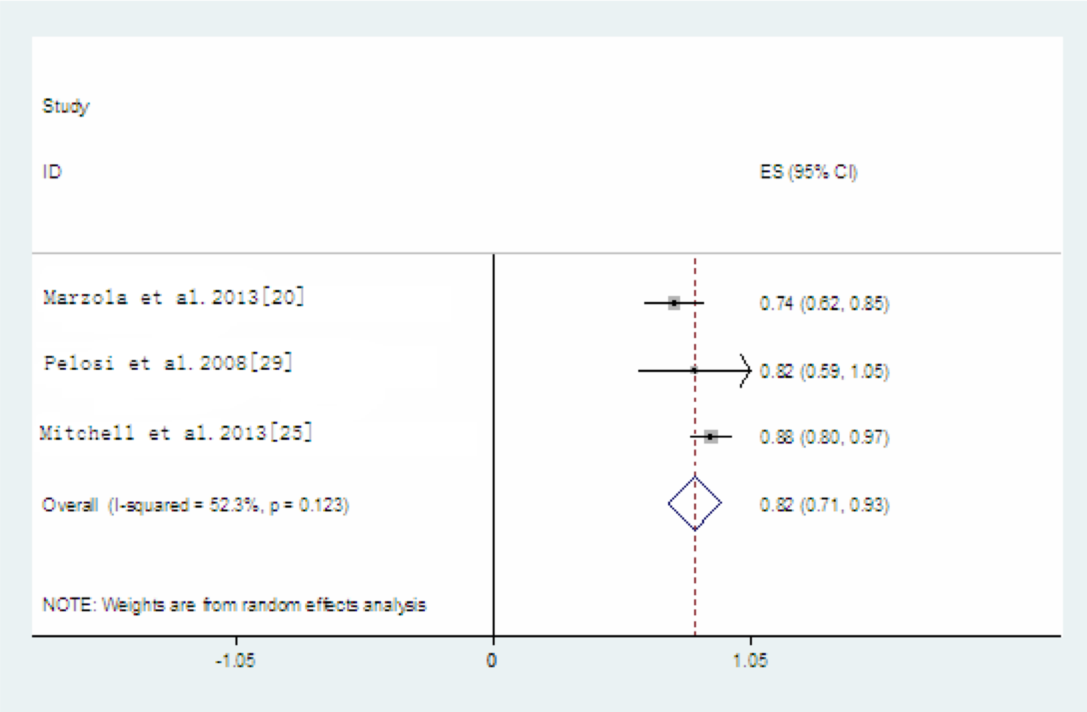

Supplementary Figure 17: Detected rate 18F-Choline PET/CT while PSA > 5 ng/ml.

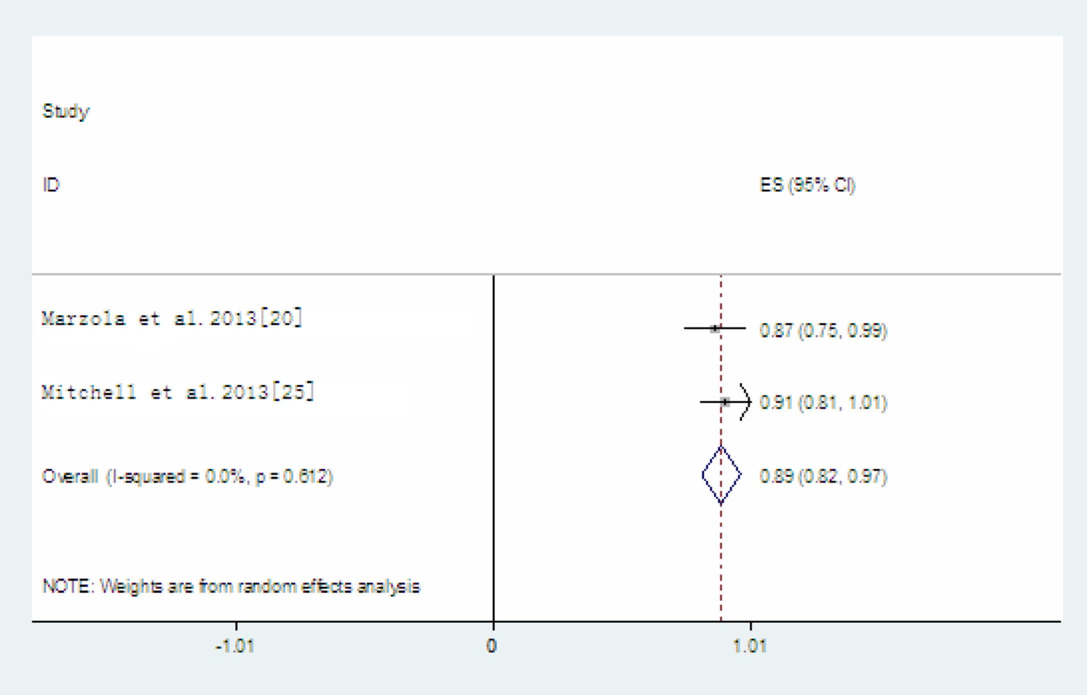

Supplementary Figure 18: Detected rate 18F-Choline PET/CT while PSA > 10 ng/ml.

Supplementary Table 1: Characteristics of studies. See_Supplementary_Table 1

Supplementary Table 2: True positive (TP), false positive (FP), true negative (TN), false negative (FN), sensitivity, specificity, positive predictive value (PPV) and negative predictive value (NPV)

Study	Radiotracer	TP	FP	FN	TN	N	sensitivity(%)	specificity(%)	PLR	NLR
Sven N et al.2008[26]	^{11}C-choline	23	2	10	14	36	70	88	5.58	0.35
Rinnab et al.2009[28]	^{11}C-choline	29	7	2	3	41	94	36	1.34	0.22
Scattoni et al.2007[30]	^{11}C-choline	19	2	0	4	25	100	66.6	2.73	0.04
Giovacchini et al.2010.[17]	^{11}C-choline	67	10	8	85	170	86.7	89.5	8.49	0.12
Giovacchini et al.2010.[18]	^{11}C-choline	145	14	26	173	358	85	93	11.33	0.16
Castellucci et al.2011[34]	^{11}C-choline	29	0	6	67	102	83	100	111.44	0.18
Jong et al.2008[36]	^{11}C-choline	10	2	0	10	22	100	83.3	4.96	0.06
Picchio et al.2003[37]	^{11}C-choline	44	3	11	42	100	80	93.3	12	0.26
Bertagna et al.2011[38]	^{11}C-choline	6	3	4	32	45	60	91	7	0.44
Rinnab et al.2007[39]	^{11}C-choline	37	7	2	4	50	94.8	36.3	1.49	0.14
Breeuwsma AJ et al.2010[40]	^{11}C-choline	57	0	13	0	70	81	100	3.63	0.48
Ceci et al.2014[41]	^{11}C-choline	109	0	9	32	150	92	100	60.73	0.08
Jong et al.2003[42]	^{11}C-choline	12	0	10	14	36	55	100	16.3	0.44
Fuccio et al.2010[43]	^{11}C-choline	19	0	3	3	25	86	100	6.78	0.17
Kitajima et al.2014[44]A	^{11}C-choline	33	2	28	24	87	54	92	7.03	0.5
Kitajima et al.2014[44]B	^{11}C-choline	45	0	5	20	70	90	100	37.47	0.11
Kitajima et al.2014[44]C	^{11}C-choline	13	1	3	78	95	81	99	64.19	0.19
Picchio et al.2012[46]	^{11}C-choline	24	1	3	50	78	89	98	45.33	0.11
Mitchell et al.2013[25]	^{18}F-choline	106	9	8	35	158	93	80	4.55	0.09
Husarik et al.2008[47]	^{18}F-choline	57	0	5	6	68	90	100	12.78	0.09
Simone et al.2015[15]	^{18}F-choline	111	2	29	4	146	78.9	76.9	2.78	0.31
Schoder et al.2005[27]	^{18}F-choline	28	3	60	0	91	32	0	0.37	5.44
Pelosi et al.2008[29]	^{18}F-choline	24	1	5	26	56	82.7	96.2	22.34	0.18
Schillaci et al.2012[31]	^{18}F-choline	33	0	3	13	49	91.7	100	25.35	0.1
Panebianco et al.2012[32]	^{18}F-choline	63	3	13	5	84	83	63	2.21	0.27
Mamede et al.2013[21]	^{18}F-choline	32	1	2	36	71	94	97	34.82	0.06
Henninger et al.2012[33]	^{18}F-choline	18	3	10	4	35	64.3	57.1	1.5	0.63
Vees et al.2007[35]	^{18}F-choline	3	2	4	2	11	43	50	0.86	1.14
Marzola et al.2009[20]	^{18}F-choline	126	3	0	104	233	100	97	30.74	0

Supplementary Table 3: QUADAS checklist for all included studies

First Author	1	2	3	4	5	6	7	8	9	10	11	12	13	14
Simone et al.2015[15]	Y	Y	Y	U	Y	N	N	Y	Y	U	U	Y	Y	Y
Giovacchini et al.2013[16]	Y	Y	Y	U	Y	N	N	Y	Y	U	U	Y	Y	Y
Giovacchini et al.2010[17]	Y	Y	Y	U	Y	N	N	Y	Y	U	U	Y	Y	Y
Giovacchini et al.2010[18]	Y	Y	Y	U	Y	N	N	Y	Y	U	U	Y	Y	Y
Chondrogiannis et al.2013[19]	Y	Y	Y	U	Y	N	N	Y	Y	U	U	Y	Y	Y
Marzola et al.2013 [20]	Y	Y	Y	U	Y	N	N	Y	Y	U	U	Y	Y	Y
Mamede et al.2013[21]	Y	Y	Y	U	Y	N	N	Y	Y	U	U	Y	Y	Y
Giovacchini et al.2012[23]	Y	Y	Y	U	Y	N	N	Y	Y	U	U	Y	Y	Y
Ceci et al.2014[24]	Y	Y	Y	U	Y	N	N	Y	Y	U	U	Y	Y	Y
Mitchell et al.2013[25]	Y	Y	Y	U	Y	N	N	Y	Y	U	U	Y	Y	Y
Sven N et al.2008[26]	Y	Y	Y	Y	Y	Y	Y	Y	Y	Y	U	Y	Y	Y
Schoder et al.2005[27]	Y	Y	Y	U	Y	N	N	Y	Y	U	U	Y	Y	Y
Rinnab et al.2009[28]	Y	Y	Y	Y	Y	Y	Y	Y	Y	Y	U	Y	Y	Y
Pelosi et al.2008[29]	Y	Y	Y	U	Y	N	N	Y	Y	U	U	Y	Y	Y
Scattoni et al.2007[30]	Y	Y	Y	Y	Y	Y	Y	Y	Y	U	U	Y	Y	Y
Schillaci et al.2012[31]	Y	Y	Y	U	Y	N	N	Y	Y	U	U	Y	Y	Y
Panebianco et al.2012[32]	Y	Y	Y	U	Y	N	N	Y	Y	U	U	Y	Y	Y
Henninger et al.2012[33]	Y	Y	Y	U	Y	N	N	Y	Y	U	U	Y	Y	Y
Castellucci et al.2011[34]	Y	Y	Y	U	Y	N	N	Y	Y	U	U	Y	Y	Y
Vees et al.2007[35]	Y	Y	Y	Y	Y	Y	Y	Y	Y	Y	U	Y	Y	Y
Jong et al.2003[36]	Y	Y	Y	Y	Y	Y	Y	Y	Y	Y	U	Y	Y	Y
Picchio et al.2003[37]	Y	Y	Y	U	Y	N	N	Y	Y	U	U	Y	Y	Y
Bertagna et al.2011[38]	Y	Y	Y	U	Y	N	N	Y	Y	U	U	Y	Y	Y
Rinnab et al.2007[39]	Y	Y	Y	Y	Y	Y	Y	Y	Y	U	U	Y	Y	Y
ANTHONIUS J et al.2010[40]	Y	Y	Y	U	Y	N	N	Y	Y	U	U	Y	Y	Y
Ceci et al.2014[41]	Y	Y	Y	U	Y	N	N	Y	Y	U	U	Y	Y	Y
Fuccio et al.2010[43]	Y	Y	Y	U	Y	N	N	Y	Y	U	U	Y	Y	Y
Kitajima et al.2014[44]	Y	Y	Y	U	Y	N	N	Y	Y	U	U	Y	Y	Y
Picchio et al.2012[46]	Y	Y	Y	U	Y	N	N	Y	Y	U	U	Y	Y	Y
Husarik et al.2008[47]	Y	Y	Y	Y	Y	Y	Y	Y	Y	U	U	Y	Y	Y
Nanni et al.2014[48]	Y	Y	Y	U	Y	N	N	Y	Y	U	U	Y	Y	Y
Cimitan et al.2006[49]	Y	Y	Y	U	Y	N	N	Y	Y	U	U	Y	Y	Y
Krause et al.2008[50]	Y	Y	Y	U	Y	N	N	Y	Y	U	U	Y	Y	Y
Castellucci et al.2009[51]	Y	Y	Y	U	Y	N	N	Y	Y	U	U	Y	Y	Y
Rinnab et al.2008[52]	Y	Y	Y	Y	Y	Y	Y	Y	Y	Y	U	Y	Y	Y
Fuccio et al.2012[53]	Y	Y	Y	U	Y	N	N	Y	Y	U	U	Y	Y	Y
Giovacchini et al.2010[54]	Y	Y	Y	U	Y	N	N	Y	Y	U	U	Y	Y	Y
Ceci et al.2013[55]	Y	Y	Y	U	Y	N	N	Y	Y	U	U	Y	Y	Y
Fuccio et al.2011[56]	Y	Y	Y	U	Y	N	N	Y	Y	U	U	Y	Y	Y
Graute et al.2012[57]	Y	Y	Y	U	Y	N	N	Y	Y	U	U	Y	Y	Y
Souvatzoglou et al.2011[58]	Y	Y	Y	U	Y	N	N	Y	Y	U	U	Y	Y	Y
Schilling et al.2008[59]	Y	Y	Y	Y	Y	Y	Y	Y	Y	U	U	Y	Y	Y
Richter et al.2010[60]	Y	Y	Y	Y	Y	Y	Y	Y	Y	Y	U	Y	Y	Y
Breeuwsma AJ et al.2012[61]	Y	Y	Y	U	Y	N	N	Y	Y	U	U	Y	Y	Y

Representative spectrum of patients. 2-Selection criteria. 3-Reference standard reliable. 4-Is the time between performance of reference standard and index test short enough?. 5-Whole or random sample received verification. 6-Same reference standard. 7-Reference standard independent of the index test. 8-Was the execution of the index test described in sufficient detail to permit replication of the test?. 9-Was the execution of the reference standard described in sufficient detail to permit replication of the test ?. 10-Were the index test results interpreted without knowledge of the results of the reference standard?. 11-Were the reference standard interpreted without knowledge of the results of the index test results?. 12-Same clinical data available. 13-Uninterruptable test result reported. 14-Withdrawals explained.
