# Supplementary material for: Trigger pSA predicting recurrence from positive choline PET/CT with prostate cancer after initial treatment
Source: Oncotarget. 2018 Jan 24;9(18):14630–41. doi: 10.18632/oncotarget.24318 (PMC5865695; doi:10.18632/oncotarget.24318)
Supplement: Supplementary file 2 [file oncotarget-09-14630-s002.docx]

Supplementary Table 1: Characteristics of studies

| First Author | | N. | Study Type | Radiotracer | Treatment | Mean Age(range)±SD | Reference standar |
| --- | --- | --- | --- | --- | --- | --- | --- |
| Simone et al.2015[15] | 146 | P | 18F | RP | 68 (63.1–72.3) | Biopsy/histology and imaging/FUP |  |
| Giovacchini et al.2013[16] | 75 | R | 11C | RP | 67(53-82) | Biopsy/imaging/FUP |  |
| Giovacchini et al.2010[17] | 170 | R | 11C | RP | n.r. | Biopsy/histology and imaging/FUP |  |
| Giovacchini et al.2010[18] | 358 | R | 11C | RP | 67(51-83) | Biopsy/imaging/FUP |  |
| Chondrogiannis et al.2013[19] | 46 | R | 18F | Radiotherapy | 71.3(51-84) | Biopsy/histology and imaging/FUP |  |
| Marzola et al.2013 [20] | 233 | R | 18F | RP | 69.7(50-87) | Biopsy/histology and imaging/FUP |  |
| Mamede et al.2013[21] | 71 | R | 18F | RP | 66.2(54-81) | Biopsy/histology and imaging/FUP |  |
| Giovacchini et al.2012[23] | 170 | R | 11C | RP | n.r. | Biopsy/histology and imaging/FUP |  |
| Ceci et al.2014[24] | 140 | R | 11C | Radiotherapy | 73(54-87) | Biopsy/histology and imaging/FUP |  |
| Mitchell et al.2013[25] | 176 | R | 18F | RP and Radiotherapy | 60.6(42-78) | Biopsy/histology and imaging/FUP |  |
| Sven N et al.2008[26] | 36 | R | 11C | RP | 68 (51–78) | Histological examination |  |
| Schoder et al.2005[27] | 91 | R | 18F | RP | 65±7 | Trus biopsy and PSA valus |  |
| Rinnab et al.2009[28] | 41 | R | 11C | RP | 64.9 (52–76) | Histological examination |  |
| Pelosi et al.2008[29] | 56 | P | 18F | RP | 67.9±7 | Biopsy/imaging/FUP |  |
| Scattoni et al.2007[30] | 25 | P | 11C | RP | 65.7±7.5 | Histological examination |  |
| Schillaci et al.2012[31] | 49 | P | 18F | RP | 70.9±7 | Biopsy/histology and imaging/FUP |  |
| Panebianco et al.2012[32] | 84 | P | 18F | RP | (56-72) | Trus biopsy and PSA valus |  |
| Henninger et al.2012[33] | 35 | R | 18F | RP | n.r. | Biopsy/histology and imaging/FUP |  |
| Castellucci et al.2011[34] | 102 | R | 11C | RP | 68(54-82) | Biopsy/histology and imaging/FUP |  |
| Vees et al.2007[35] | 11 | P | 18F | RP | 62(54-67) | Histological examination |  |
| Jong et al.2003[36] | 22 | P | 11C | RP or Radiotherapy | 68±9 | Histological examination |  |
| Picchio et al.2003[37] | 100 | P | 11C | RP or Radiotherapy | 70.52(45-81) | Biopsy/histology and imaging/FUP |  |
| Bertagna et al.2011[38] | 45 | R | 11C | RP or Radiotherapy | 70±7 | histological prostate site mapping |  |
| Rinnab et al.2007[39] | 50 | R | 11C | RP or Radiotherapy | 65.9(52-79) | Histological examination |  |
| ANTHONIUS J et al.2010[40] | 80 | P | 11C | Radiotherapy | n.r. | Biopsy/histology and imaging/FUP |  |
| Ceci et al.2014[41] | 150 | R | 11C | RP or Radiotherapy | 69.6±6.8 | Biopsy/histology and imaging/FUP |  |
| Fuccio et al.2010[43] | 25 | R | 11C | RP | 70.2 (58–80) | Biopsy/histology and imaging/FUP |  |
| Kitajima et al.2014[44] | 87 | R | 11C | RP | 66(49-87) | Biopsy/histology and imaging/FUP |  |
| Picchio et al.2012[46] | 78 | R | 11C | RP | 69(47-82) | Biopsy/histology and imaging/FUP |  |
| Husarik et al.2008[47] | 68 | P | 18F | RP or Radiotherapy | 66.4 | Histological examination |  |
| Nanni et al.2014[48] | 18 | P | 18F | RP | 67.9 ± 6.4 | Biopsy/histology and imaging/FUP |  |
| Cimitan et al.2006[49] | 100 | R | 18F | RP or Radiotherapy | (49–81) | Biopsy/imaging/FUP |  |
| Krause et al.2008[50] | 63 | R | 11C | RP or Radiotherapy | 69 (45–83) | Biopsy/histology and imaging/FUP |  |
| Castellucci et al.2009[51] | 190 | R | 11C | RP | 68 (54–83) | Biopsy/histology and imaging/FUP |  |
| Rinnab et al.2008[52] | 15 | P | 11C | RP or Radiotherapy | 62.1(53-73) | Histological examination |  |
| Fuccio et al.2012[53] | 123 | R | 18F | RP | 67.6(54–83) | Biopsy/histology and imaging/FUP |  |
| Giovacchini et al.2010[54] | 109 | R | 11C | RP | 66.4±6.2 | Biopsy/histology and imaging/FUP |  |
| Ceci et al.2013[55] | 157 | R | 11C | RP | 70(50-85) | Biopsy/histology and imaging/FUP |  |
| Fuccio et al.2011[56] | 14 | R | 11C | RP | 67(55-82) | Biopsy/histology and imaging/FUP |  |
| Graute et al.2012[57] | 82 | R | 18F | RP | 67.1±7.0 | Biopsy/imaging/FUP |  |
| Souvatzoglou 2011[58] | 37 | R | 11C | RP | 64 (49–78) | Biopsy/histology and imaging/FUP |  |
| Schilling et al.2008[59 | 10 | R | 11C | RP or Radiotherapy | n.r. | Histological examination |  |
| Richter et al.2010[60] | 73 | P | 11C | RP | 65.62(41-78) | Histological examination |  |
| Breeuwsma AJ et al. 2012[61] | 64 | P | 11C | RP | n.r. | Biopsy/histology and imaging/FUP |  |
|  |  |  |  |  |  |  |  |

RP radical prostatectomy, nr not recorded, N. number of patients, FUP follow-up, 11C ^11^C-choline, 18F ^18^F-choline
